# Corporate Social Responsibility in Public Health During the COVID-19 Pandemic: Quarantine Hotel in China

**DOI:** 10.3389/fpubh.2021.620930

**Published:** 2021-02-02

**Authors:** Xiaodong Teng, Yi-Man Teng, Kun-Shan Wu, Bao-Guang Chang

**Affiliations:** ^1^Intelligent Accounting Research Center, Shandong University of Finance and Economics, Accounting School, Jinan, China; ^2^College of Modern Management, Yango University, Fuzhou, China; ^3^Department of Business Administration, Tamkang University, Taipei, Taiwan; ^4^Department of Accounting, Tamkang University, Taipei, Taiwan

**Keywords:** COVID-19, quarantine hotel, public health, corporate social responsibility, marketing strategies

## Introduction

The outbreak of a novel coronavirus pneumonia, known as COVID-19, started in Wuhan, China, and spread rapidly and widely around the globe. The COVID-19 pandemic has had a major impact on the well-being of people and countries around the world, with major implications for public health, society, safety and the economy (1, 2). As a result, hotels, viewed as a declining industry, have suffered a staggering drop in average occupancy rates (3). The hospitality industry is “*already facing collapse*” and is “*fighting for survival*,” as a result of the pandemic.

As positive cases of COVID-19 escalate, hospitals face problems with overcrowding and insufficient isolation space (4). In addition, scholars propose that those patients suspected to have COVID-19 and patients with COVID-19 experiencing only mild symptoms should prevent interaction with other household members by isolating themselves in a hotel (5). The hotel, coordinated by a tertiary referral hospital and attended the preventive medicine regulations, not only as a public health resource for the containment of COVID-19 but also as more than a place for quarantining in Madrid (6). Further, there is increasing worry from frontline healthcare workers (HCW) about contracting the virus and contaminating loved ones (7). To prevent the transmission of the virus and protect parents and/or children from the epidemic, HCW and suspected COVID-19 patients, need temporary quarantine from their families. A quarantine hotel is a possible solution to address this demand for temporary quarantine accommodation. Prior literatures also discussed about the role of quarantine hotels associated with the use of preventive protocols in hospitals to reduce the occurrence of hospital outbreaks. The authors reveal the hospitals and hotels have common characteristics, such as individual rooms and toilets, which made the quarantine hotels not only a viable solution for the containment of COVID-19, but for the caring of patients and HCWs (6, 8). Quarantine hotels are a community-based public health intervention designed to mitigate the spread of COVID-19 within the community.

With the COVID-19 virus sweeping the world and disrupting lives, livelihoods, and communities, and putting enormous strain on public health as a whole, “corporate social responsibility” must now play its part. Stakeholder theory has also been actively applied in the theory and practice of “corporate social responsibility” (9). “Corporate social responsibility” refers to “*the responsibility of enterprises for their impacts on society*” (10). Today, “corporate social responsibility” is the new tool for hoteliers when they encounter an irresistible force. Especially for the time of necessitating an urgent search of the external factors. Thus, focusing on “corporate social responsibility” is now a significant and effective way to not only solve current problems, but also assist with the future strategic decisions of tourism enterprises and the tourism industry as a whole during the COVID-19 crisis (11).

As we write this paper, the coronavirus is ongoing. Considering its novelty, few studies have studied its impact on “corporate social responsibility.” Moreover, there are few studies that have examined this epidemiological catastrophe from the “corporate social responsibility” in public health perspective. In this article, preliminary ideas are discussed on how the quarantine hotel has demonstrated “corporate social responsibility” by assisting Chinese communities, particularly the HCW, and its potential implications on marketing strategies during and after the pandemic.

## The Quarantine Hotel is an Adjusted Operations Model Which Also Provides a Model for “Corporate Social Responsibility”

“Corporate social responsibility” is often used as a comprehensive term to describe a variety of issues relating to the responsibilities of business (12). “Corporate social responsibility” has developed into a new global governance model to promote the ability to reach collective decisions on transnational subjects (13). The COVID-19 crisis has tested companies on their commitment to ethical business conduct and “corporate social responsibility” (14). For example, some companies have donated funds, personal protective equipment, hand sanitizer, respiratory systems and testing kits to hospitals.

As a result of the COVID-19 pandemic and the impact of new challenges to the hotel industry, hoteliers adjusted their operations to overcome obstacles, such as redundancies. Post-pandemic accomplishment over fighting the virus will build an organization's genuine “corporate social responsibility” and provide a more superior relationship with the general public. Previous hotel industry literature actively promotes their “corporate social responsibility” efforts and focusses on the perceptions of the hospitality industry's environmental practices (15). Now, the quarantine hotel is not only an important ally in the fight against the COVID-19 pandemic, it also provides another “corporate social responsibility” within the public health model.

## “Corporate Social Responsibility” Initiatives of the Quarantine Hotel

How will stakeholders be involved in the quarantine hotel's “corporate social responsibility” activities and what does it mean to them?

### Medical HCW

Due to the high contagiousness of COVID-19, healthcare professionals are occupationally exposed to high biological risk (16). The protection of health care professionals is of great concern and importance during the COVID-19 pandemic (17). In Italian, for Infection control and provided a specific early detection programme, HCW staffs can be hosted safely in hotel facilities during the COVID-19 pandemic (8). Taking care of the mental health of quarantine employees is a critical part of the public health response and is also a “corporate social responsibility” (18). Quarantine hotels provide their premises to house medical staff when dealing with COVID-19 patients. Supporting HCW in all aspects of health and safety is important to sustain a healthy workforce and is pivotal in protecting them against the virus. A report discovered that the provision of rest areas and basic physical needs, such as food, resulted in greater satisfaction among HCW (19).

In order to prevent transmission of the virus and protect their parents or children from the pandemic, some HCW have temporarily quarantined themselves from their family. The quarantine hotels provided the much-needed private space to rest or self-isolate, making life a little less stressful for those battling COVID-19.

### Travelers or Residents From Abroad

Governments worldwide have enforced that all travelers or residents returning from overseas must be quarantined in designated accommodation (quarantine hotels) for 14 days from the day they arrive in the country. Hoteliers are helping to prevent the spread of COVID-19 by complying with the quarantine order and providing travelers and residents with accommodation for the 14-day quarantine period. As a result, there is now a large proportion of travelers and residents living in quarantine hotels.

### Hotel Employees

The hospitality industry supports millions of livelihoods; however, job losses have resulted from the devastating impact of the COVID-19 pandemic. Providing quarantine space is a smart business move, as quarantine packages will provide some economic relief for the hospitality industry. As a quarantine hotel, the adjusted operations model guarantees staff will keep their jobs and ensures wages will still be paid.

Staff of quarantine hotels will also receive extra medical education to identify microbiological characteristics and perform diagnosis, disinfection and self-protection technology. They are also trained in operation guides, complying with anti-epidemic and disinfection standards, and implementing quarantine services. Quarantine hotels will help organizations navigate workforce shifts through agile workforce strategies.

Although the roles of the hotel staff have been adjusted, their new skills will prevail. Underlying core skills, along with new skills in medical education, anti-epidemic and disinfection standards are the new currency and will be the key to building resilient workforces in the future. Moving forward, it will provide staff with the additional skills and new mindsets needed to obtain high-demand jobs in either health care or other businesses. For example, kitchen and cleaning staff could move on to work in an elderly care center, long-term care facilities and/or nursing homes. Thus, hoteliers are also helping staff achieve long-lasting workforce resilience.

### Shareholders and Investors of the Quarantine Hotel

As the COVID-19 pandemic continues, more countries require both citizens and foreign visitors arriving from abroad to enter a mandatory 14-day isolation period at a quarantine hotel. Hence, there is an increasing demand for this facility. In Malaysia, anyone traveling into the country will have to pay the full cost, US$34.50 a day, for their compulsory quarantine hotel. In Taiwan, in addition to the income from the quarantine travelers, as a designated quarantine hotel the hotelier will also receive some compensation from the government. Offering quarantine packages slightly relieves the financial pressures caused by the COVID-19 outbreak, which will benefit shareholders and investors.

### Government

By offering quarantine accommodation during the COVID-19 pandemic, the hospitality industry is working with the government to support their quarantine requirements policy. This solves the problem of tourists requiring quarantine accommodation and also minimizes potential leaks in the government's outbreak prevention efforts.

## Marketing Strategies in the Hospitality Industry During and After the Pandemic

The public health crisis caused by the COVID-19 pandemic provides a significant opportunity for the hospitality industry to actively participate in various “corporate social responsibility” initiatives. Mid-pandemic, as the hospitality industry introduces the quarantine hotel, their “corporate social responsibility” quickly adopts the market-driven strategy to control, educate and manage the basic services in demand. The designated quarantine hotel is a short-term “corporate social responsibility” strategy in the fight against the COVID-19 pandemic. It also potentially accelerates a new era of development of “corporate social responsibility” in the long-term marketing strategy.

This article implicates that some short-term marketing strategies that change consumer habits can lead to a long-term marketing philosophy. First, enhance the insertion of robotics technology and artificial intelligence (AI) into the hotel industry. The increased emphasis on physical social distancing and decreasing personal contact are significant measures to reduce transmission of diseases. The quarantine hotel provides three meals a day via services that avoid contact with guests. In the future, hotels can apply AI and robotics technology to positions such as bellboy, busboy and room service to avoid personal contact, while still maintaining service standards.

Second, because the COVID-19 virus can spread through contact with contaminated surfaces, cleanliness and hygiene are paramount (20). Hoteliers should focus their attention on hygiene and cleanliness and promote this message via social media e.g., “*We have high standards of cleanliness and hygiene*.” As the quarantine hotel employees have been trained and educated on essential preparatory and prevention measures, ranging from hygiene measures that includes increased frequency of cleaning and sanitizing, to guidelines on how to handle suspected or confirmed cases of COVID-19 and implementing quarantine services, customers will feel comfortable and safe during their stay at the hotel.

## Conclusion

The present paper is an opinion article which studies the impact of COVID-19 on “corporate social responsibility” within public health from the quarantine hotel perspective. The goal of this opinion article is to enrich the understanding of COVID-19's impact on corporate social responsibility in public health, and to suggest avenues for quarantine hotels' actions from social enterprise perspective, during and post COVID-19 in order to mitigate its effects. Academically, the contribution of this study is to examine the impact of COVID-19 on corporate social responsibility within public health, this paper will help broaden the scope of corporate social responsibility research in the COVID-19 crisis and provide some insights for hoteliers. Thus, this study provides a pioneer reference for similar studies in the future.

From a practical implications point of view, the outbreak of COVID-19 has disturbed the daily operation and even survival of hotels worldwide. The quarantine hotel volunteered to house HCW or those requiring quarantine, which is a positive CSR way for hotels to open their doors and give back to society during this public health crisis. This demonstrates altruism and egoism, which is a win-win corporate social responsibility marketing strategy. While COVID-19 drives the hotel industry's CSR strategy in the short term, researchers need to explore and discover effective marketing strategies which will bring beneficial corporate social responsibility to their stakeholders in the long term.

After the COVID-19 pandemic, what are the opportunities and challenges for corporate social responsibility in the long term? Post-pandemic, researchers should strive to reinforce the theory and epistemology of the hospitality industry to help them become more resilient and attain effective post-disaster recovery. Hospitality practitioners and scholars should carefully investigate the empirical influence of the COVID-19 crisis and seek to improve hoteliers' corporate social responsibility, and a new era marketing strategy for hospitality industry operations from the stakeholders' perspective. In future research, scholars should include in-depth interviews, surveys, or a mixed-methods approach to research to gather data on the topics raised in related articles.

## Author Contributions

All authors listed have made a substantial, direct and intellectual contribution to the work, and approved it for publication.

## Conflict of Interest

The authors declare that the research was conducted in the absence of any commercial or financial relationships that could be construed as a potential conflict of interest.
